# Correlations of severity of asthma in children with body mass index, adiponectin and leptin

**DOI:** 10.1002/jcla.22915

**Published:** 2019-05-31

**Authors:** Chunli Ma, Yujun Wang, Man Xue

**Affiliations:** ^1^ Department of Pediatrics Baoji People's Hospital Baoji China; ^2^ Department of Integrated Traditional Chinese and Western Medicine Children's Hospital Affiliated to Xi'an Jiaotong University (Xi'an Children's Hospital) Xi’an China

**Keywords:** adiponectin, asthma, body mass index, leptin

## Abstract

**Background:**

This study aims to investigate the correlations of asthma in children with body mass index (BMI), adiponectin, and leptin.

**Methods:**

A total of 122 children with asthma in our hospital from January 2017 to February 2018 were randomly selected and divided into control group (normal) and observation group (BMI > 28 kg/m^2^) according to BMI. BMI, adiponectin, and leptin levels between the two groups were measured and compared, and correlations of disease grade with BMI, adiponectin, or leptin were analyzed. Moreover, risk factors for asthma in children were also identified.

**Results:**

Body mass index, leptin level, forced vital capacity (FVC), FVC%, and forced expiratory volume in 1s (FEV1)/FVC in observation group were significantly higher than those in control group (*P* < 0.05), while the adiponectin level, forced expiratory capacity in 1s (FEC1), and FEV1% in observation group were significantly lower than those in control group (*P* < 0.05). The amount of severe patients in observation group was much larger than that in control group. The severity of disease was positively correlated with BMI and leptin and negatively correlated with adiponectin. BMI, adiponectin, and leptin were identified as risk factors for asthma in children.

**Conclusion:**

Adiponectin, leptin, and BMI are involved in the pathogenesis of asthma in children, suggesting they might be therapeutic targets for clinical treatment.

## 1 INTRODUCTION

Asthma in children is generally found with a high prevalence rate, the incidence rate of which, according to statistical analysis, increases year by year with the environmental changes. Once asthma occurs, children often suffer from long‐term cough and chest distress, bringing great economic burden to the whole family.^1^, ^2^, ^3^ At present, there have been few clinical studies on asthma in children, and its pathogenesis remains poorly understood.^4^, ^5^, ^6^ Clinical research has revealed that obesity plays a critical role among several pathogenic factors of asthma. The body mass index (BMI) of patients is considered as an important indicator to the severity of the disease.^7^, ^8^, ^9^ Adipocytes in the body can produce adipokines, adiponectin, and leptin, which may be related to the pathogenesis of asthma.^10^, ^11^ To investigate the pathogenesis of asthma in children, the correlations of asthma in children with BMI, adiponectin, and leptin were mainly investigated in this study.

## 2 MATERIALS AND METHODS

### 2.1 General data

A total of 122 children patients complicated with asthma in our hospital from January 2017 to February 2018 were randomly selected and divided into control group (n = 60, normal weight, BMI < 28 kg/m^2^), including 32 males and 28 females aged (11.56 ± 3.01) years on average, and observation group (n = 62, BMI > 28 kg/m^2^), including 33 males and 29 females aged (11.90 ± 3.07) years on average, according to BMI. Patients in both groups met the diagnostic criteria for asthma in children. The study was approved by the ethics committee of Baoji People's Hospital, and informed consents were signed by the patients’ guardians.

Inclusion criteria: children patients meeting the diagnostic criteria for asthma and children patients without severe injury in vital organs, such as heart, liver, and kidney.

Exclusion criteria: children patients taking hormone drugs within 2 weeks before the experimental study, children patients with the clinical symptom of infection, or children patients with severe allergic reactions.

### 2.2 Methods

About 3 mL fasting venous blood was drawn from each patient. After 5 minutes, serum was collected by centrifugation, which was then cryopreserved in a cryogenic refrigerator.

Determination of BMI: BMI = body weight (kg)/height (m)^2^. It was measured for a total of three times, and the average was calculated.

Determination of pulmonary function indexes: Forced expiratory volume in 1s (FEV1) and forced vital capacity (FVC) were determined using the pulmonary function instrument. FVC% and FEV1% were predicted, and FEV1/FVC was calculated.

Determination of adiponectin: The content of adiponectin in fasting venous serum was measured via enzyme‐linked immunosorbent assay using the adiponectin kit.^12^

Determination of leptin: Leptin was determined via radioimmunoassay, and the leptin kit was placed in a radioimmune counter for reading.^13^

The number of children patients in different disease grades in both groups was recorded, and the disease grade was divided into mild, moderate, and severe. Mild: Asthma symptoms in patients affected the activity and sleep. Asthma occurred more than twice in the evening every month and less than once per week on average. FEV1 ≥ 80% of predicted value or PEF ≥ 80% of personal optimum value. The mutation rate of PEF or FEV1 was 20%‐30%. Moderate: There were asthma symptoms in patients in the daytime affecting the activity and sleep, asthma occurred more than once in the evening per week. FEV1 = 60%‐79% of predicted value or PEF = 60%‐79% of personal optimum value. The mutation rate of PEF or FEV1 > 30%. Severe: Asthma symptoms occurred frequently in the daytime every day and often occurred in the evening. FEV1 < 60% of predicted value or PEF < 60% of personal optimum value. The mutation rate of PEF or FEV1 > 30%.

### 2.3 Observation indexes

Adiponectin, leptin, and pulmonary function indexes (FEV1, FVC, FVC% and FEV1%) in each patient were determined.

### 2.4 Data statistics and analysis

Statistical Product and Service Solutions (SPSS) 25.0 software was used for data statistical analysis. Measurement data were presented as ($\overset{¯}{X}$mean ± standard deviation), and student *t* test was used. Enumeration data were presented as case (n), and chi‐square test was adopted. Pearson correlation analysis was performed among variables. *P* < 0.05 suggested that the difference was statistically significant.

## 3 RESULTS

### 3.1 Comparisons of BMI, adiponectin, leptin, and pulmonary functions

BMI, leptin level, FVC, FVC%, and FEV1/FVC in observation group were significantly higher than those in control group (*P* < 0.05), while the adiponectin level, FEC1, and FEV1% in observation group were significantly lower than those in control group (*P* < 0.05; Table 1).

**Table 1:** Comparisons of BMI, adiponectin, leptin, and pulmonary functions between the two groups of patients ($\overset{¯}{X}$±*s*)

Index	Control group	Observation group
BMI (kg/m^2^)	24.69 ± 2.15	30.05 ± 2.66*
Adiponectin (μg/mL)	15.1 ± 1.03	2.9 ± 0.11*
Leptin (ng/mL)	5.98 ± 0.45	19.05 ± 1.56*
FVC (L)	3.51 ± 0.22	4.11 ± 0.31*
FVC% (%)	101.6 ± 6.11	108.9 ± 5.98*
FEC1 (L)	3.96 ± 0.29	2.65 ± 0.16*
FEV1/FVC	70.5 ± 4.51	90.6 ± 5.06*
FEV1% (%)	85.6 ± 5.11	68.9 ± 3.67*

* *P* < 0.05 vs control group

### 3.2 Comparison of disease grade

**Figure 1:**
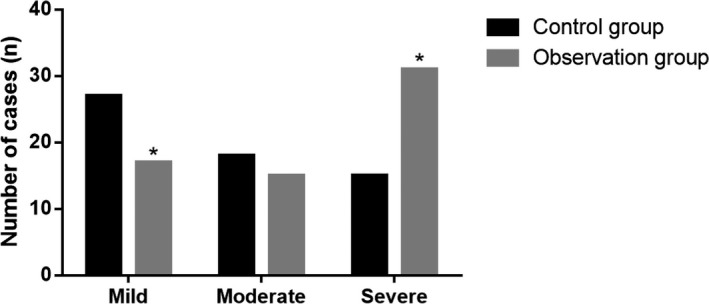
Comparison of disease grade between the two groups of patients. *P < 0.05 vs control group

In observation group, there were 17 mild patients, 15 moderate patients, and 31 severe patients. In control group, there were 27 mild patients, 18 moderate patients, and 15 severe patients. The number of severe patients in observation group was obviously more than that in control group, while the number of mild patients in observation group was obviously fewer than that in control group (*P* < 0.05; Figure 1).

### 3.3 Correlations of grade of asthma in children with BMI, adiponectin, and leptin

**Figure 2:**
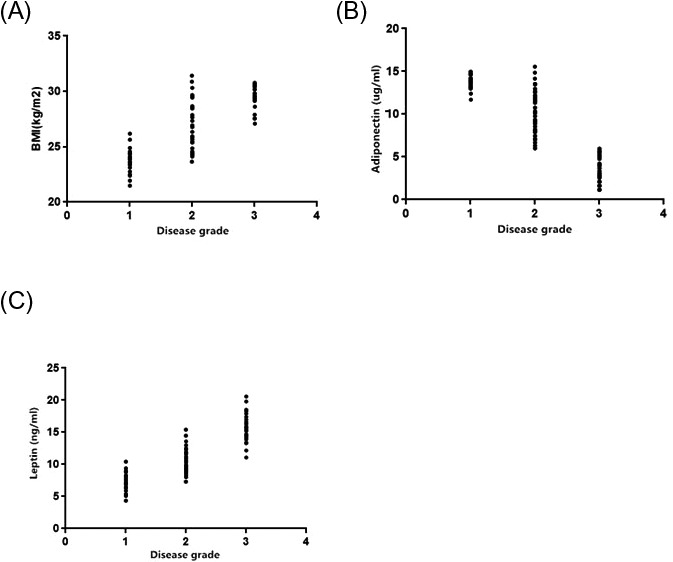
Correlation analysis. A, between grade and BMI. B, between disease grade and adiponectin. C, between disease grade and leptin

Correlations of disease grade with BMI, adiponectin, and leptin were analyzed, and it was found that the disease grade was positively correlated with BMI (*r* = 0.8621, *P* < 0.0001) and leptin (*r* = 0.8901, *P* < 0.0001), and negatively correlated with adiponectin (*r *= −0.9203, *P* < 0.0001; Figure 2).

### 3.4 Logistic regression analyses of risk factors for asthma in children

Risk factors for asthma in children were analyzed, and it was found that BMI, leptin, adiponectin, and blood pressure were identified as risk factors (Table 2).

**Table 2:** Logistic regression analyses of risk factors for asthma in children

Factors	*p*	OR	95% CI
Gender	0.068	1.313	0.267, 1.452
BMI	0.016	0.891	0.381, 1.601
Leptin	0.012	1.056	0.276, 1.311
Adiponectin	0.030	1.302	0.263, 1.452
Blood pressure	0.036	1.012	0.321, 2.356
Age	0.086	1.006	0.165, 1.308

## 4 DISCUSSION

According to clinical statistics, the number of the obese increases year by year with changes in people's living habit. There are a variety of complications in patients induced by obesity, in which asthma is a major one.^14^ Once asthma occurs, it will bring much inconvenience to the patients’ life and work. Moreover, obesity has not only a greater impact on adults, but also a close correlation with asthma in children.^15^ Clinical studies have showed that asthma in children is related to the environment and climate changes, indicating a close correlation with the body system. If there are symptoms of obesity in children patients, asthma will be aggravated and the risk of complications will be increased.^16^ Therefore, the correlation between BMI and asthma was investigated in this study, so as to understand the exact relationship between obesity and asthma.

In this study, BMI and leptin level in observation group were significantly higher than those in control group, while the adiponectin level was significantly lower than that in control group. The number of severe child patients in observation group was obviously higher than that in control group, suggesting that BMI is implicated with severity of the asthma. Gastroesophageal reflux occurs easily in obese children, in which food will reflow into the airway to stimulate the vagus nerves in respiratory tract in different degrees, thus leading to bronchospasm easily. Studies have also found that if acidic food reflows into the airway, there will be bronchial hyperresponsiveness. Due to the excessive accumulation of fat in the soft tissue around the upper respiratory tract of obese patients, intensive driving force is needed when the air is exhaled out of the body through the airway, and such driving force will result in airway wall vibration, thereby leading to asthma.^17^

In this study, it was found that the adiponectin level was lower in observation group than that in control group. Adiponectin was negatively correlated with the disease grade, and adiponectin was one of the risk factors for asthma in children. Adipose tissues secrete a variety of adipokines, including pro‐inflammatory adipokines and anti‐inflammatory adipokines, which are involved in various physiological function regulations in the body.^18^ Adiponectin is a kind of endogenous anti‐inflammatory bioactive polypeptide secreted by adipocytes, which regulates oxidative stress and resists inflammation in the body. It is revealed that adiponectin produces asthma symptoms mainly through the oxidative stress pathway, and its declining level will aggravate the asthma.^19^ In this study, it was found that the leptin level in observation group was obviously lower than that in control group, leptin was positively correlated with the disease grade, and leptin was one of the risk factors for asthma in children. Leptin is also a kind of adipokine, and it has a correlation with lipid reserve in the body, which maintains the normal body weight via regulating energy intake and consumption. If the leptin level significantly declines, the sensitivity of hypothalamus to the fat will be reduced, resulting in the increase of fat intake and thus gaining of body weight. The excessive secretion of leptin in the body induces asthma symptoms through inhibiting the immune T‐cell function. Besides, leptin can promote the production of leukotrienes, increase the secretion of immune factor immunoglobulin E, and elevate the expression of inflammatory mediators, thus leading to airway inflammatory response.^20^ The shortcoming of this study was that our preliminary data should be further validated with a large number of samples and the exact mechanism pathway of adiponectin and leptin to exacerbate the asthma has not been determined, and it will be the emphasis in subsequent research.

In conclusion, asthma in children is associated with BMI, adiponectin, and leptin. The high value of BMI and leptin, along with low level of the adiponectin, indicates severe asthma in patients. BMI, adiponectin, and leptin have certain predictive values for the condition of asthma, suggesting they might be used as potential indicators for future treatment.
